# Analysis of sugar crystal size in honey

**DOI:** 10.1016/j.mex.2022.101823

**Published:** 2022-08-18

**Authors:** Mareike Weber, Mario Meixner, Reinhard Dasbach, Wilfried Rozhon, Margot Dasbach

**Affiliations:** a Department of Agriculture, Ecotrophology, and Landscape Development, Anhalt University of Applied Sciences, Bernburg, Saxony-Anhalt 06406, Germany; b Am Provianthaus 7, Bernburg, Saxony-Anhalt 06406, Germany

**Keywords:** Honey, Glucose, Microscopy, Crystallisation, Mouth feel, Sugar crystal size

## Abstract

Honey consists typically of more than 80% sugars, predominantly fructose and glucose. Glucose-rich honey crystallizes more rapidly than honey with a high fructose content. However, the size of the sugar crystals is crucial for the mouth feel of crystallised honey. Honeys containing small crystals have a creamy consistency, which is preferred by most consumers. In contrast, large crystals cause a coarse mouth feel. Factors affecting crystal size are of vital interest for the production of high-quality honey and thus analysis of sugar crystal size in honey is crucial. Here we present a simple and efficient method for measuring the size of sugar crystals in honey. A honey drop is placed on a coverslip, which is centrifuged using a converted smoothie maker. This spreads the drop over the coverslip and separates the sugar crystals from each other. Subsequently, the size of the crystals can be conveniently measured by microscopy. Compared to squeezing the honey drop between slide and coverslip, this approach avoids the risk of breaking the crystals. Moreover, the method is highly reproducible as indicated by intra-day and inter-day standard deviations of 7 to 14% for crystal sizes.

Simple method for preparation of honey for crystal size analysis by microscopy.

Use of cheap, easily accessible equipment.

High intra and inter-day reproducibility.

## Graphical abstract

**Figure:**
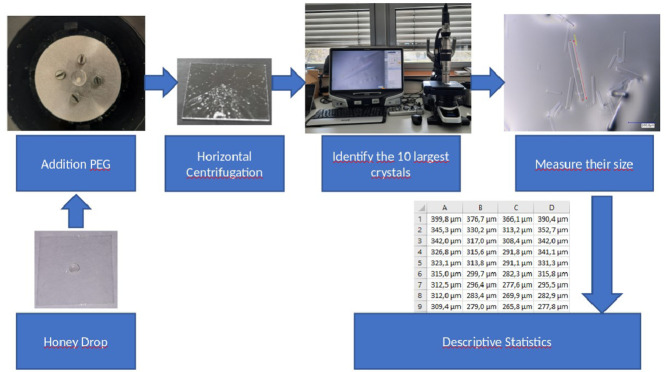

**Specifications table**

**Table:** 

Subject area:	Agricultural and Biological Sciences
More specific subject area:	Crystal size analysis
Method name:	Analysis of sugar crystal size in honey
Name and reference of original method:	Costa et al. [2] Brazilian Journal of Food Technology 18, 155–161. DOI: 10.1590/1981-6723.7314 Dettori et al. [5] LWT - Food Science and Technology 95, 333–338. DOI: 10.1016/j.lwt.2018.04.092 Quintero-Lira et al. (2017) European Food Research Technology 234, 619-626. DOI: 10.1007/s00217-016-2775-0
Resource availability:	NA

## Method details

### Background

Honey consists typically of more than 80% (w/w) sugars and less than 20% (w/w) water. Other compounds are crucial for colour and taste but are present in total at less than 1% (w/w). Amongst the sugars, the monosaccharides fructose and glucose are present in high quantities of typically more than 30% (w/w) while other sugars including the oligosaccharides maltose, isomaltose, turanose, trehalose and raffinose are only present in small quantities [6]. The composition of the sugars, particularly the ratio of fructose and glucose, depends mainly on the forage flowers of bees. Consequently, the fructose to glucose ratio can vary in a wide range from approximately 0.4 to 2.5 [3,^7^,8]. Fructose-rich honey remains usually liquid for several months or years. In contrast, crystallisation of glucose-rich honey often begins shortly after harvesting, as glucose is significantly less soluble in water than fructose; solubility of glucose: 47 g/100 g water [14]; solubility of fructose: 385 g/100 g water [15]. Importantly, the crystallisation conditions determine the size of the sugar crystals [4,^11^,13], which can reach a size of more than 200 µm in length [11]. Most consumers prefer honey with small crystals since it has a creamy mouth feel [12] while honey with large crystals is sensed as coarse grained. Measures to influence the creaminess of honey are therefore intensively investigated [2,^5^,^9^,^11^,12]. This, however, requires simple and reliable methods for crystal size determination in honey. Currently, the crystal size is mainly measured by microscopic analysis [2,^5^,10] and, in a few cases, by laser diffraction [2]. In principle, microscopic analysis of crystal size is straightforward and simple. However, due to its high sugar content honey is highly viscous, which complicates uniform spreading of the crystals on a microscopic slide. Simple spread out, although frequently used [5,10], is only successful with honey samples containing a few crystals per gram. In order to disperse more crystallised samples strong forces would be required, which would break particularly large crystals and thereby prevent accurate crystal size analysis.

Here we describe a simple method for determining the crystal size in honey by microscopy. A drop of honey is placed on a coverslip, covered with polyethylene glycol 200 and centrifuged using a converted smoothie maker. This spreads the honey sample over of the coverslip and removes the polyethylene glycol 200. Subsequently, the coverslip is placed upside up on a microscopic slide and the crystal size is determined by microscopic analysis. This method has the advantage that the crystals are well distributed over the slide without application of high forces and the thickness of the honey film is thin enough preventing overlapping of the crystals.

### Materials and reagents

Polyethylene glycol 200 (Carl Roth Ph.Eur.)
Coverslip 18 × 18 mm
Piston pipettes, 1–10 µl and 10–100 µl
Micro spatula
Analytical balance (readout at least 0.1 mg)

### Instrumentation

**Fig. 1:**
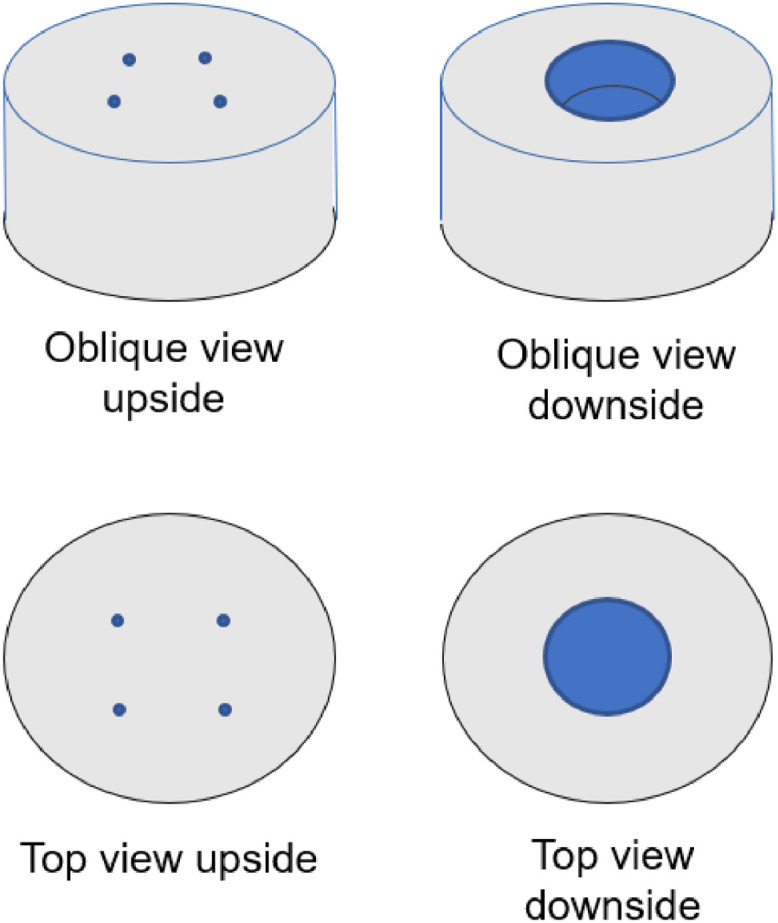
Aluminium cylinder for conversion of the smoothie maker.

**Fig. 2:**
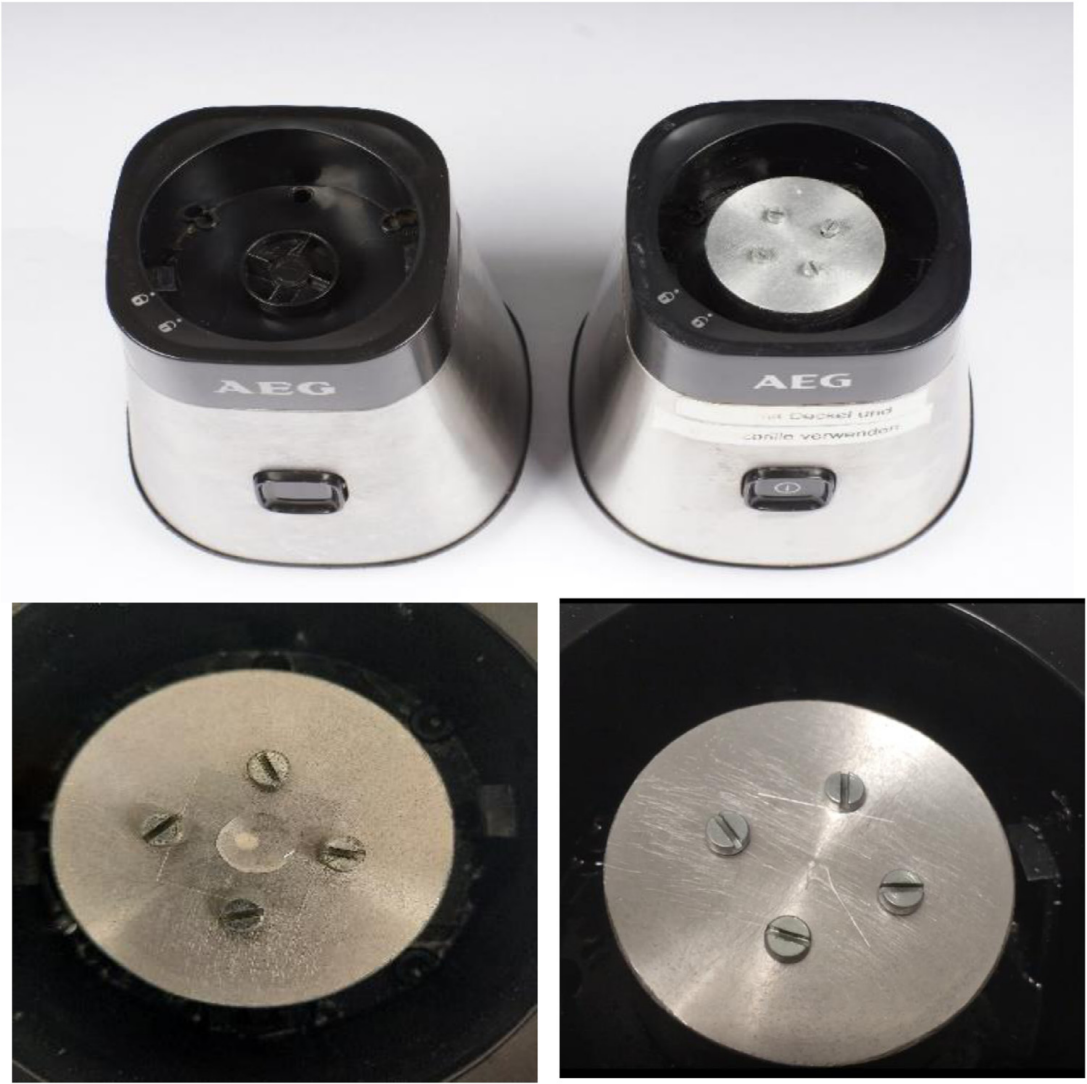
Top: Smoothie maker before (left) and after the adaption for coverslip centrifugation; below: cover slip with honey drop and PEG before (left) and after (right) centrifugation

A smoothie-maker (AEG MiniMixer SB 2700, Electrolux, Stockholm, Sweden, 23,000 rpm) was converted to a coverslip centrifuge by cutting four 10 mm deep threads into an aluminium cylinder of 48 mm in diameter and 23 mm width to accept M3 metal screws (Fig. 1). A whole with a diameter of 25 mm and a depth of 8 mm depth was milled on the underside of the cylinder to accommodate the wheel of the smoothie maker. The aluminium cylinder was fixed to the wheel of the smoothie maker using two-component adhesive (Fig. 2). Using the screws, a coverslip can be clamped to the cylinder for centrifugation.

For microscopic analysis a microscope with at least 250x magnification and equipped with a camera should be used. In this study a VHX-7000N digital microscope (Keyence, Osaka, Japan) with 3D modus was used.

### Protocol

#### Sample preparation by centrifugation

1. Place a dry and fat-free 18 mm coverslip on an analytical balance and tare.
2. Transfer 1.0±0.2 mg honey onto the coverslip using a micro spatula. Try to place the drop into the centre of the coverslip.
3. Fix the coverslip in the converted smoothie maker with the screws.
4. Cover the honey drop with 50 µl of polyethylene glycol 200. *Note: The polyethylene glycol decreases the viscosity of the honey.*
5. Within the next minute start the smoothie maker for 20 s to centrifuge the sample briefly. *Note 1: Sample preparation is depictured in Supplementary* Fig. 1 *and a video showing the procedure is available in the Supplementary Materials section. Note 2: In this step the honey drop is spread over the coverslip and a homogeneous honey film is achieved. Honey crystals are not overlapping. With too short centrifugation the crystals are not well separated (Supplementary* Fig. 2a*). In contrast, excessive centrifugation leads to crystal conglomeration (Supplementary* Fig. 2c *and* 2d*).*

### Microscopy

1. Remove the coverslip from the converted smoothie maker and place it upside up on a slide.
2. Inspect the whole coverslip for sugar crystals using a microscope in 3D mode at 250x magnification for sugar crystals and document them using the camera. *Note: Multiple pictures from different focus levels are combined to a single picture with extended depth of field.*
3. Depending on the application, measure either the size distribution of the crystals or the sizes of the 10 largest crystals per sample. *Note 1: Since mainly large crystals are responsible for the mouth feel of honey it is often sufficient to evaluate the ten largest crystals per slide rather than measuring the whole size distribution. However, the most suitable way depends of course on the application. Note 2: To make sure to identify the 10 largest crystals inspect the whole cover slip and subsequently measure at least 30 huge crystals and use the size of the 10 largest ones.*
4. For each honey sample at least three coverslips should be prepared and the crystal sizes determined.

### Method validation

**Fig. 3:**
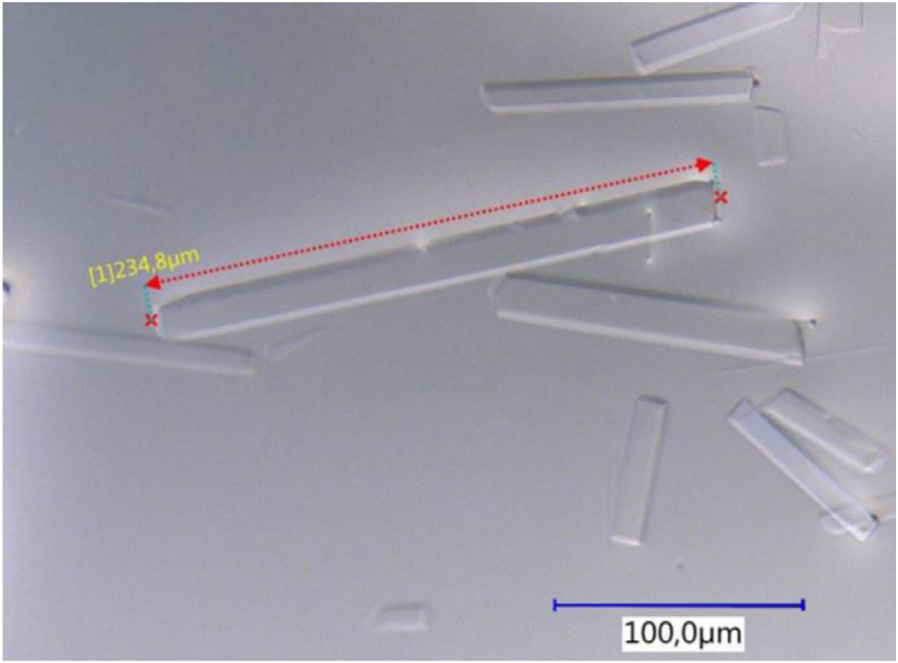
Typical micrograph of a honey sample prepared for microscopy by centrifugation. For this experiment spring flower honey was used.

The method described above allows simple and reproducible spreading of honey samples over the coverslip (Fig. 3).

**Fig. 4:**
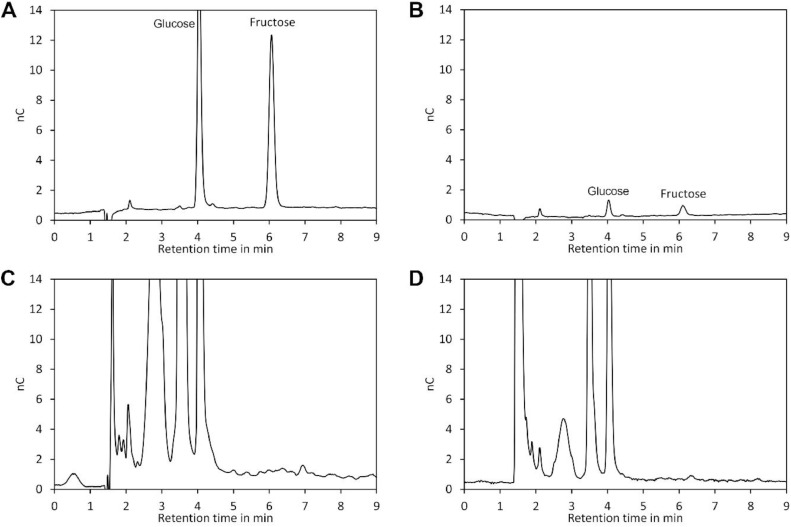
Centrifugation of the coverslip does not cause detectable sample loss. (**A**) Chromatogram of a standard containing 1 mg/l glucose and fructose separated by ion chromatography using a Carbo-PAC PA100 4 × 250 mm column and an eluent consisting of 100 mM sodium hydroxide at a flow rate of 1 ml/min. For detection an amperometric cell equipped with a gold working electrode and a silver/silver chloride (3 M KCl) reference electrode was used. (**B**) Standard with a concentration of 0.05 mg/l. (**C**) Extract of the cotton swab before centrifugation of the sample. (**D**) Extract of the cotton swab after centrifugation of a honey sample placed on a coverslip. nC, nanocoulomb.

In contrast to spreading samples between slides and coverslips, centrifugation is a gentle procedure for distributing crystals over the coverslip. However, centrifugation may pose the risk of sample loss due to centrifugal forces. To investigate potential sample loss, the smoothie maker was thoroughly cleaned to prevent presence of any traces of sugars inside of the centrifugation chamber from previous experiments. Subsequent the interior surface was rubbed off with a wetted cotton swab, which was subsequently placed in 3 ml distilled water for 10 min to dissolve any sugar crystals that might have been collected by the cotton swab. Analysis by ion chromatography with pulsed amperometric detection (Fig. 4A and 4B) confirmed absence of any fructose. Next, a spring flower honey sample was centrifuged as described above. The spring flower honey had a fructose content of 37.9±0.4% (w/w) and a glucose content of 36.3±0.7% (w/w) as determined by HPLC using a Nucleosil 100-5 NH2 250 × 4 mm column and 80% acetonitrile as eluent [1]. After centrifugation, the interior of the smoothie maker was again rubbed off with a wetted cotton swab, which was placed in 3 ml distilled water for 10 min prior analysis. As shown in Fig. 4D, no fructose indicative for sample loss could be detected. This experiment was repeated six times and in all measurements fructose was below the limit of detection. The ion chromatography system used had a sensitivity of 0.05 mg/l (limit of detection) for fructose, which would even allow detection of a single fructose particle with a length of 250 µm and a width of 20 µm dissolved in 3 ml water. In contrast to fructose, glucose could not be measured using this system because compounds leached from the cotton swab interfered with the glucose peak (Fig. 4A and 4C). Nevertheless, this result clearly demonstrates that there is no detectable sample loss during centrifugation.

Having established that the method is suitable for uniform spreading of sugar crystals present in honey samples on a coverslip, we investigated the intra- and inter-day reproducibility of the methods. For that purpose, the same honey sample as used above was analysed in quadruplicate (each time with three technical repeats) on four consecutive days (Table 1). These data confirm that the developed method is highly reproducible with inter-day and intra-day relative standard deviations (RSD) in the range of approximately 7–14%.

**Table 1:** Intra- and inter-day repeatability for determination of crystal length (largest 10 crystals each sample).

Experiment	Repeats	Crystal length		
		Average [µm]	SD [µm]	RSD [%]
Day 1	4	312.6	33.1	10.6
Day 2	4	276.1	19.1	6.9
Day 3	4	320.7	44.3	13.8
Day 4	4	305.8	25.0	8.2
Inter-day	16	303.8	36.0	11.9

## Conclusion

Preparation of honey for microscopic analysis can be challenging due to its high viscosity. Here we show that centrifugation using an adapted smoothie maker is a simple, rapid and gentle method for spreading honey over a cover slip. Subsequently, crystal size is determined by microscopic analysis. The method shows high reproducibility with an intra-day RSD of 7–14% and an inter-day RSD of 12%.

## Conflict of interest

The authors declare that they have no known competing financial interests or personal relationships that could have appeared to influence the work reported in this paper.

## Data Availability

Data will be made available on request.

## Appendix Supplementary materials

Image, video 1

Image, application 2

Image, application 3
