# Supplementary material for: Analysis of sugar crystal size in honey
Source: MethodsX. 2022 Aug 18;9:101823. doi: 10.1016/j.mex.2022.101823 (PMC9440417; doi:10.1016/j.mex.2022.101823)
Supplement: Supplementary file 2 [file mmc2.pdf]

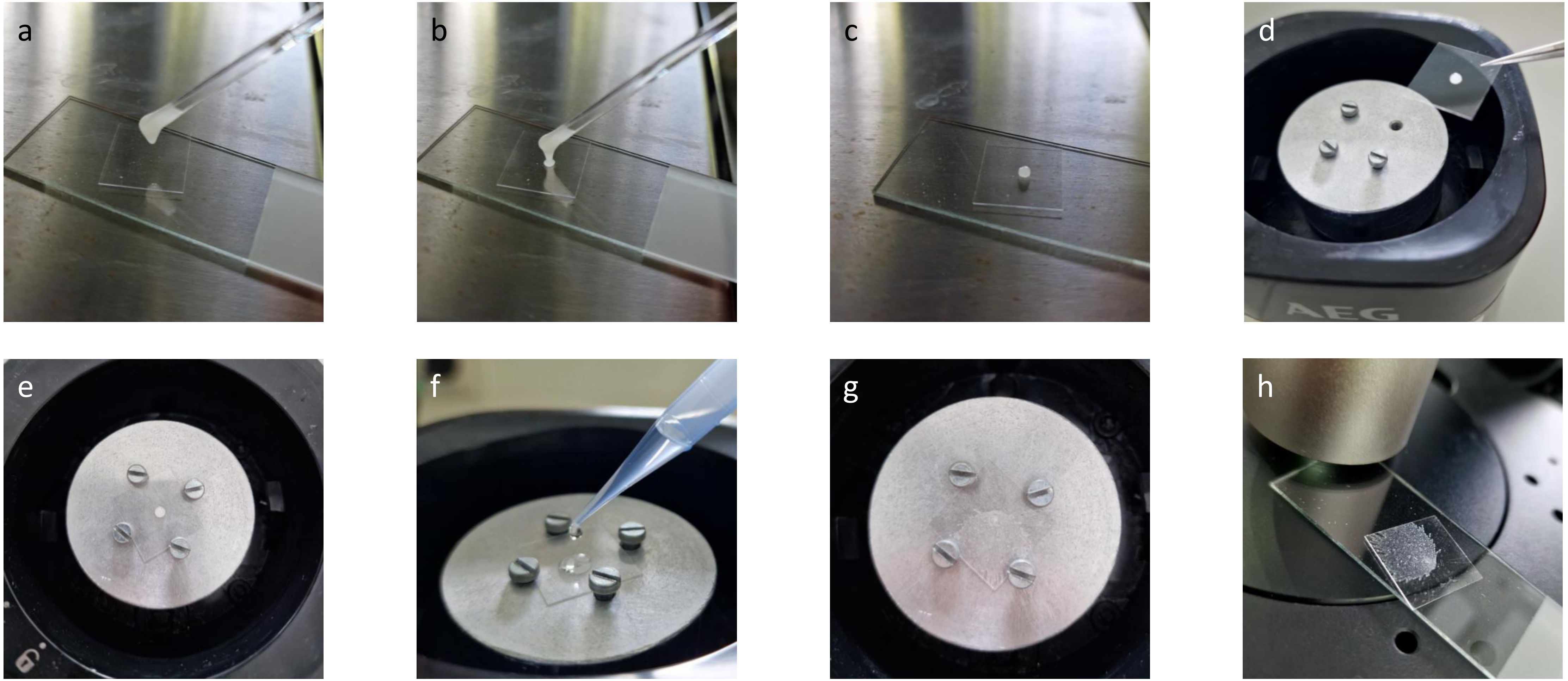

Supplementary Figure 1: Sample preparation for microscopic analysis by horizontal centrifugation.
a-c) Placing the honey drop on the cover slip. d-e) Clamping the coverslip on the cylinder. f) Adding 50 µl of PEG 200. g) Cover slip after centrifugation. h) Placing the cover slip upside up on a slide for microscopic analysis.
