# Supplementary material for: Analysis of sugar crystal size in honey
Source: MethodsX. 2022 Aug 18;9:101823. doi: 10.1016/j.mex.2022.101823 (PMC9440417; doi:10.1016/j.mex.2022.101823)
Supplement: Supplementary file 3 [file mmc3.pdf]

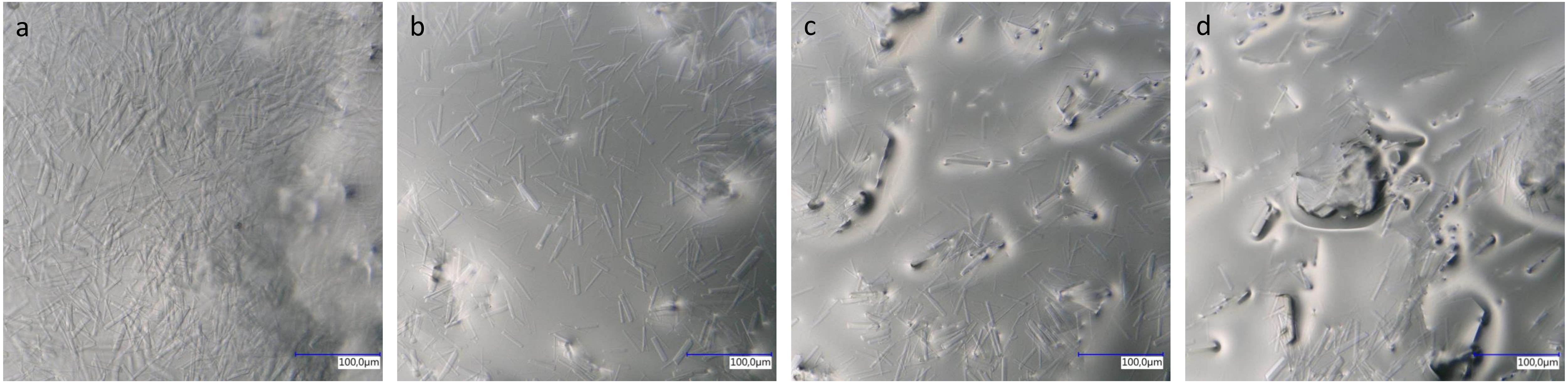

Supplementary Figure 2: Impact of centrifugation time on spreading of crystals. Micrographs of honey drops centrifuged for a) 5 seconds b) 20 seconds c) 60 seconds d) 120 seconds.
